# Supplementary material for: Graded recruitment of pupil-linked neuromodulation by parametric stimulation of the vagus nerve
Source: Nat Commun. 2021 Mar 9;12:1539. doi: 10.1038/s41467-021-21730-2 (PMC7943774; doi:10.1038/s41467-021-21730-2)
Supplement: Supplementary file 1 — Supplementary Information [file 41467_2021_21730_MOESM1_ESM.pdf]

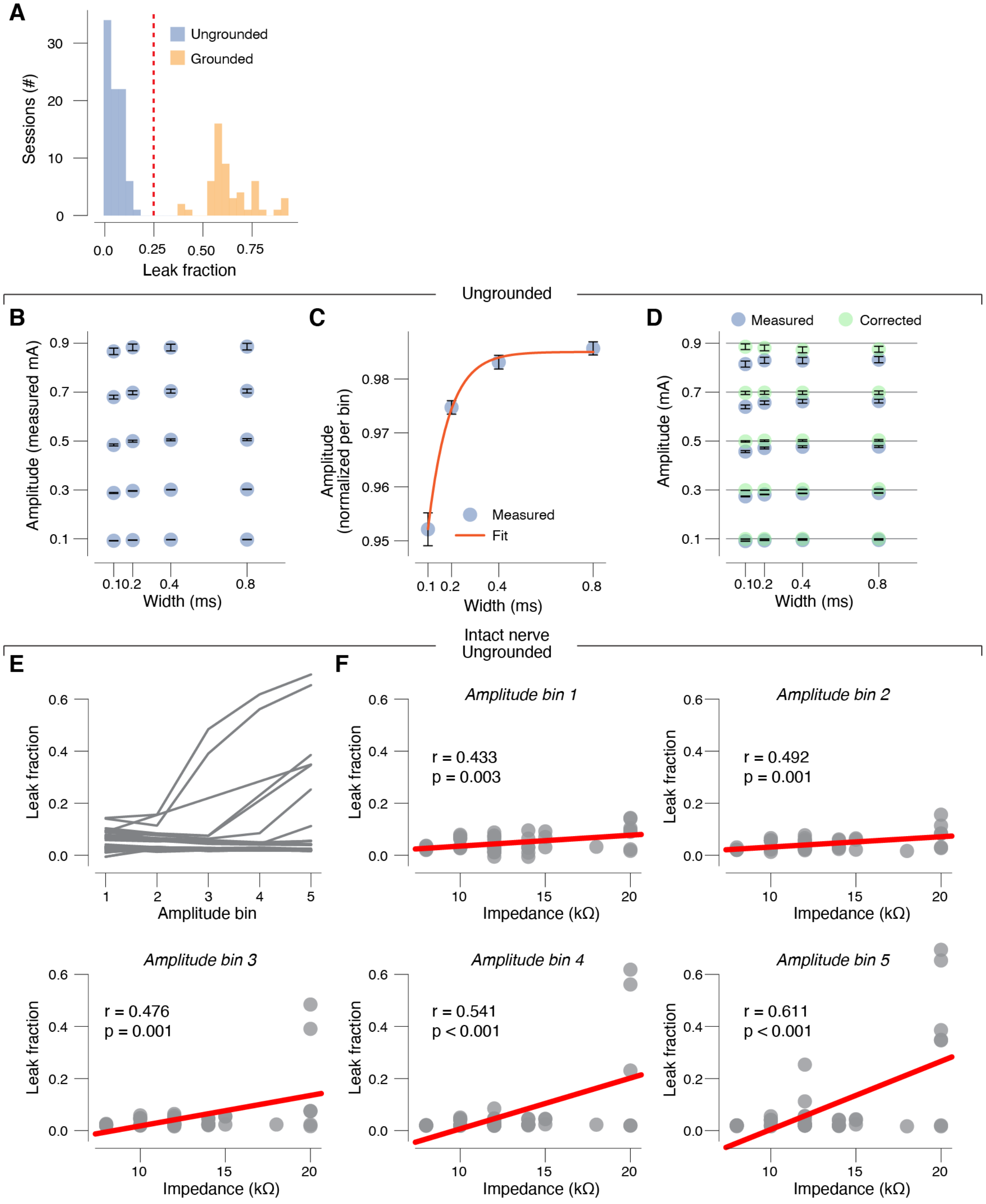

Supplementary Figure 1. (A) Histogram of session-wise observed leak fraction (extent of current loss for the smallest amplitude pulses with the largest two pulse widths; see Methods). **(B)** Measured pulse amplitudes for five amplitude and four width bins, averaged across train rates. Data are presented as mean values ± s.e.m. (across sessions). All sessions in ungrounded conditions are included (intact, single cut, and double cut nerve). **(C)** As B, but collapsed across amplitude bins. Red line, fitted sigmoid (see Methods). **(D)** As B, but including amplitude measures that were corrected for leak and filtering (green symbols). Black horizontal lines indicate total applied current. **(E)** Observed leak fraction for each amplitude bin

(averaged across highest two pulse widths and all train rates). Each line is a session. **(F)** Relationship between observed leak fraction and measured cuff electrode impedance. Each panel is for the indicated pulse amplitude bin. Each data point is a session; red lines are fitted 1st order polynomial; stats are Pearson correlation. Panels B-D: N=18 (total of 83 sessions). Panels E-F: N=10 (total of 45 sessions).

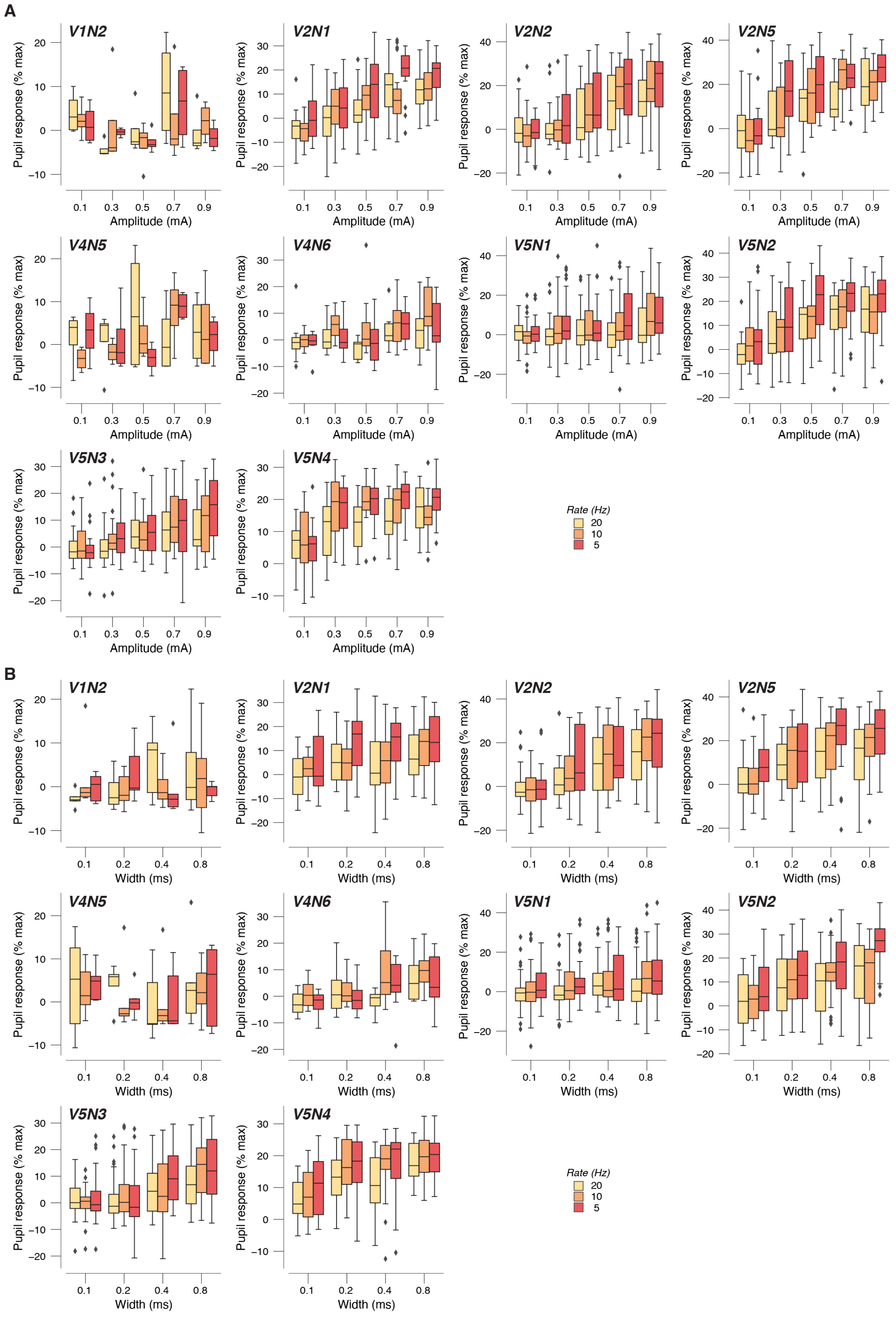
A
B
V1N2
V2N1
V2N2
V2N5
V4N5
V4N6
V5N1
V5N2
V5N3
V5N4
Pupil response (% max)
Amplitude (mA)
Width (ms)
Rate (Hz)
20
10
5

Supplementary Figure 2. (A) VNS-evoked pupil response scalars plotted separately per animal, and separately for pulse amplitudes and rates (collapsed across widths). Box plots indicate the median (center line), first quartiles (box edges), minimum/maximum values (whiskers), and outliers (diamonds; based on interquartile range). **(B)** As A, but for pulse widths and rates (collapsed across amplitudes). Source data are provided as a Source Data file.

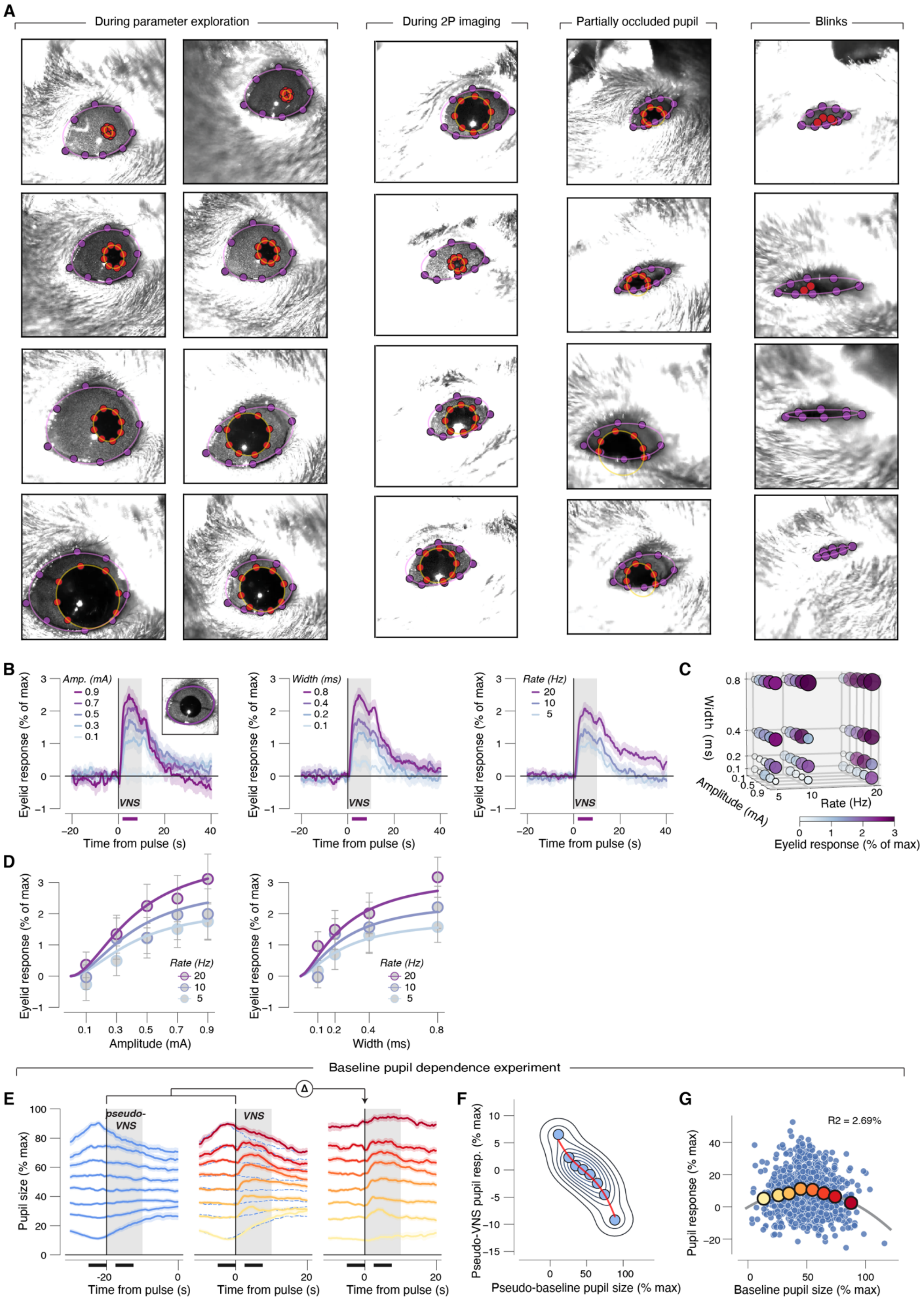

A
During parameter exploration
During 2P imaging
Partially occluded pupil
Blinks
B
Eyelid response (% of max)
Amp. (mA)
0.9
0.7
0.5
0.3
0.1
VNS
Time from pulse (s)
Width (ms)
0.8
0.4
0.2
0.1
Rate (Hz)
20
10
5
C
Width (ms)
Amplitude (mA)
Rate (Hz)
Eyelid response (% of max)
D
Amplitude (mA)
Width (ms)
Baseline pupil dependence experiment
E
Pupil size (% max)
pseudo-VNS
VNS
Δ
Time from pulse (s)
F
Pseudo-VNS pupil resp. (% max)
Pseudo-baseline pupil size (% max)
G
R2 = 2.69%
Pupil response (% max)
Baseline pupil size (% max)

Supplementary Figure 3. (A) Example frames labeled with DeepLabCut (Methods). Purple dots, eyelid labels. Red dots, pupil labels. Only labels with a likelihood bigger than 0.1 are plotted. These examples were selected to illustrate the various challenges to pupil and eye lid fitting which include large variation in size (columns 1-2), partial occlusion of the pupil by the lid (column 3) and blinks (column 4). **(B)** VNS-evoked exposed eye area response time-courses separately for each pulse amplitude (left), width (middle) and rate (right), collapsed across two other stimulation parameters. Grey window, 10 second VNS train; purple bars, interval for VNS-evoked exposed eye area scalar measures (see Methods); data are presented as mean values ± s.e.m. (across VNS events). **(C)** VNS-evoked exposed eye area scalar measure for all 60 unique parameter combinations. Response magnitude is indicated by circle size and color. **(D)** VNS-evoked exposed eye area scalars plotted separately for pulse amplitudes and rates (collapsed across widths, left) and separately for pulse widths and rates (collapsed across amplitudes, right). Colored lines, fitted log-logistic functions (Methods); data are presented as mean values ± 1.96 × s.e.m. (across VNS events). **(E)** Left: Pupil size time-courses in the 30 seconds before each VNS stimulus train, sorted into eight bins of pseudo-baseline pupil size (mean pupil size from -25 to -20 seconds before each VNS). These sorted traces illustrate that spontaneous pupil fluctuations exhibit a strong tendency to revert to the mean. Middle: same as at left, but for pupil size time-course from 10 seconds before to 20 seconds after VNS. Blue dashed lines reproduce the spontaneous fluctuations shown at left. Note the VNS-evoked responses ride on top of the general tendency of reversion to the mean. Right: difference (subtraction) of the middle and left panels, shifted to their appropriate baselines. Note the several seconds-long VNS-evoked dilation seen from mid-size baseline, but not large or small baseline. Data are presented as mean values ± s.e.m. (across [pseudo-]VNS events). **(F)** Relationship between pseudo-VNS pupil responses and pseudo baseline pupil size (panel A, left). Red line, fitted cubic function (Methods). **(G)** Relationship between VNS-evoked pupil responses, after correction for reversion to the mean (Methods) as a function of pre-VNS baseline pupil size. A 1st-order (linear) fit was superior to a constant fit ($F_{1,847} = 1.50$, $p = 0.221$) and a 2nd-order (quadratic) fit was superior to the first-order fit ($F_{1,847} = 12.67$, $p < 0.001$) (sequential polynomial regression; Methods). Grey line, fitted 2nd order polynomial (Methods). Panels B-D: N=45 repetitions for each unique parameter combination (before artefact rejection; Methods). Panels E-G: total of N=1380 repetitions of the same parameter combination (before artefact rejection; Methods). Source data are provided as a Source Data file.

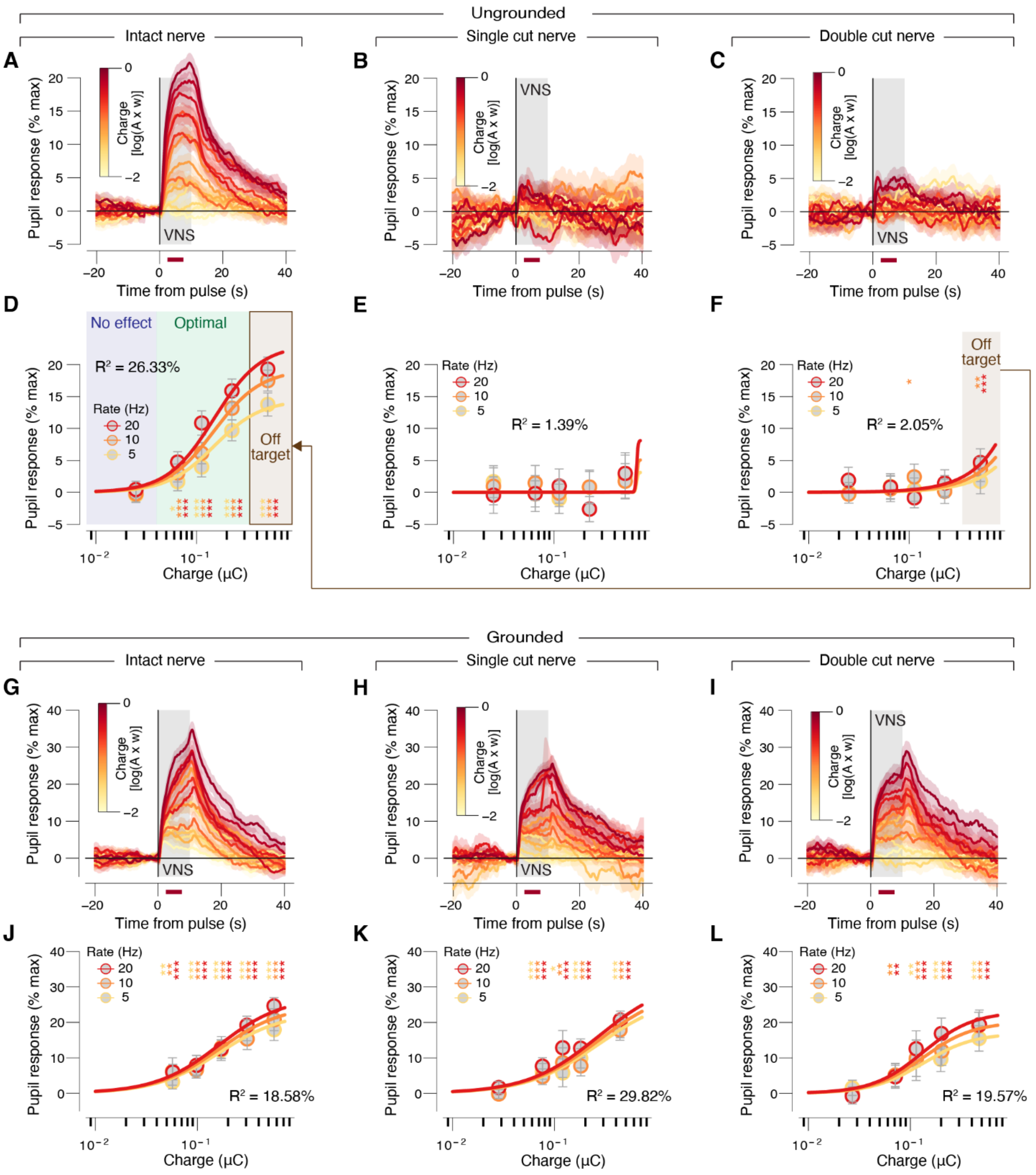

Supplementary Figure 4. (A) VNS-evoked pupil time-courses, for ungrounded animals with intact nerve. Each trace is a charge/pulse bin and train rate. Grey window, VNS train; red bar, interval for VNS-evoked pupil response scalar measures (see Methods); data are presented as mean values ± s.e.m. (across VNS events). N=45 repetitions for each unique parameter combination (before artefact rejection; Methods). **(B)** As A, but for single cut nerve. N=14 repetitions for each unique parameter combination (before artefact rejection; Methods). **(C)** As A, but for double cut nerve. N=24 repetitions for each unique parameter combination (before artefact rejection; Methods). **(D)** VNS-evoked pupil response scalar measures separately per charge/pulse bin and train rate, for ungrounded animals with intact nerve. Colored lines,

fitted log logistic function (Methods); data are presented as mean values ± 1.96 × s.e.m. (across VNS events; stats, two-sided one sample t-test (tested against 0; ***$p < 0.001$, **$p < 0.01$; *$p < 0.05$, corrected for false discovery rate). **(E)** As D, but for single cut nerve. **(F)** As D, but for double cut nerve. **(G-L)** As A-F, but for grounded animals. N=22, N=17 and N=14 repetitions for each unique parameter combination, respectively (before artefact rejection; Methods). Source data are provided as a Source Data file.

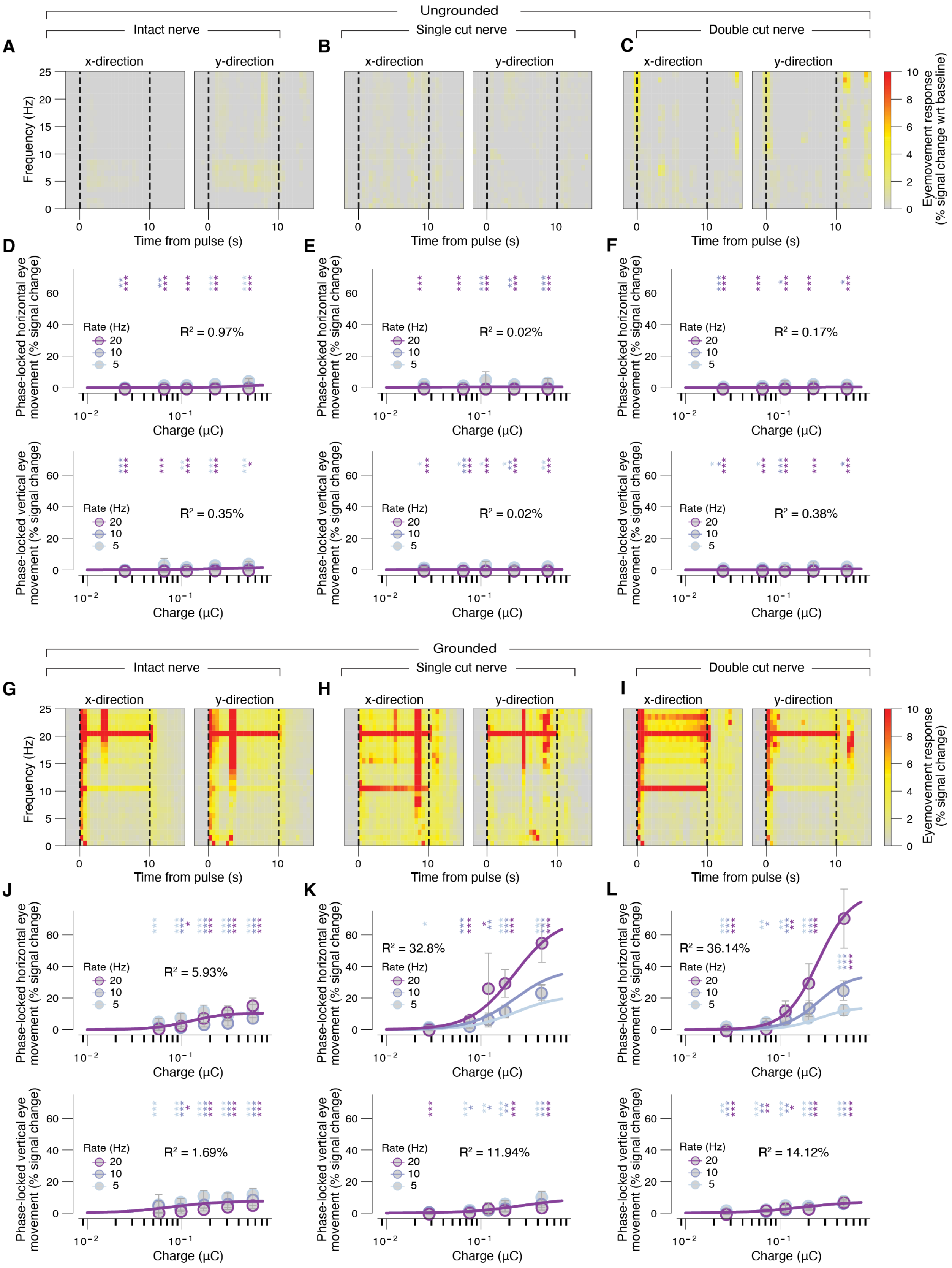
Ungrounded
Intact nerve
Single cut nerve
Double cut nerve
A
B
C
x-direction
y-direction
Frequency (Hz)
Time from pulse (s)
Eyemovement response (% signal change wrt baseline)
D
E
F
Phase-locked horizontal eye movement (% signal change)
Phase-locked vertical eye movement (% signal change)
Rate (Hz)
20
10
5
R² = 0.97%
R² = 0.02%
R² = 0.17%
R² = 0.35%
R² = 0.02%
R² = 0.38%
Charge (μC)
Grounded
G
H
I
Eyemovement response (% signal change)
J
K
L
R² = 5.93%
R² = 32.8%
R² = 36.14%
R² = 1.69%
R² = 11.94%
R² = 14.12%

Supplementary Figure 5. (A) Time-frequency representation of VNS-evoked eye movement responses, expressed as % signal change from baseline (Methods), for ungrounded animals with intact nerve. N=45 repetitions for each unique parameter combination (before artefact rejection; Methods). **(B)** As A, but for single cut nerve. N=14 repetitions for each unique parameter combination (before artefact rejection; Methods). **(C)** As A, but for double cut nerve. N=24 repetitions for each unique parameter combination (before artefact rejection; Methods). **(D)** VNS-evoked phase-locked horizontal (top) and vertical (bottom) eye movement response measures (Methods) separately per charge/pulse bin and train rate. Colored lines, fitted log logistic function (Methods); stats, two-sided one sample t-test (tested against 0; ***$p < 0.001$, **$p < 0.01$; *$p < 0.05$, corrected for false discovery rate); data are presented as mean values ± 1.96 × s.e.m. (across VNS events). **(E)** As D, but for single cut nerve. **(F)** As D, but for double cut nerve. **(G-L)** As A-F, but for grounded animals. N=22, N=17 and N=14 repetitions for each unique parameter combination, respectively (before artefact rejection; Methods). Source data are provided as a Source Data file.

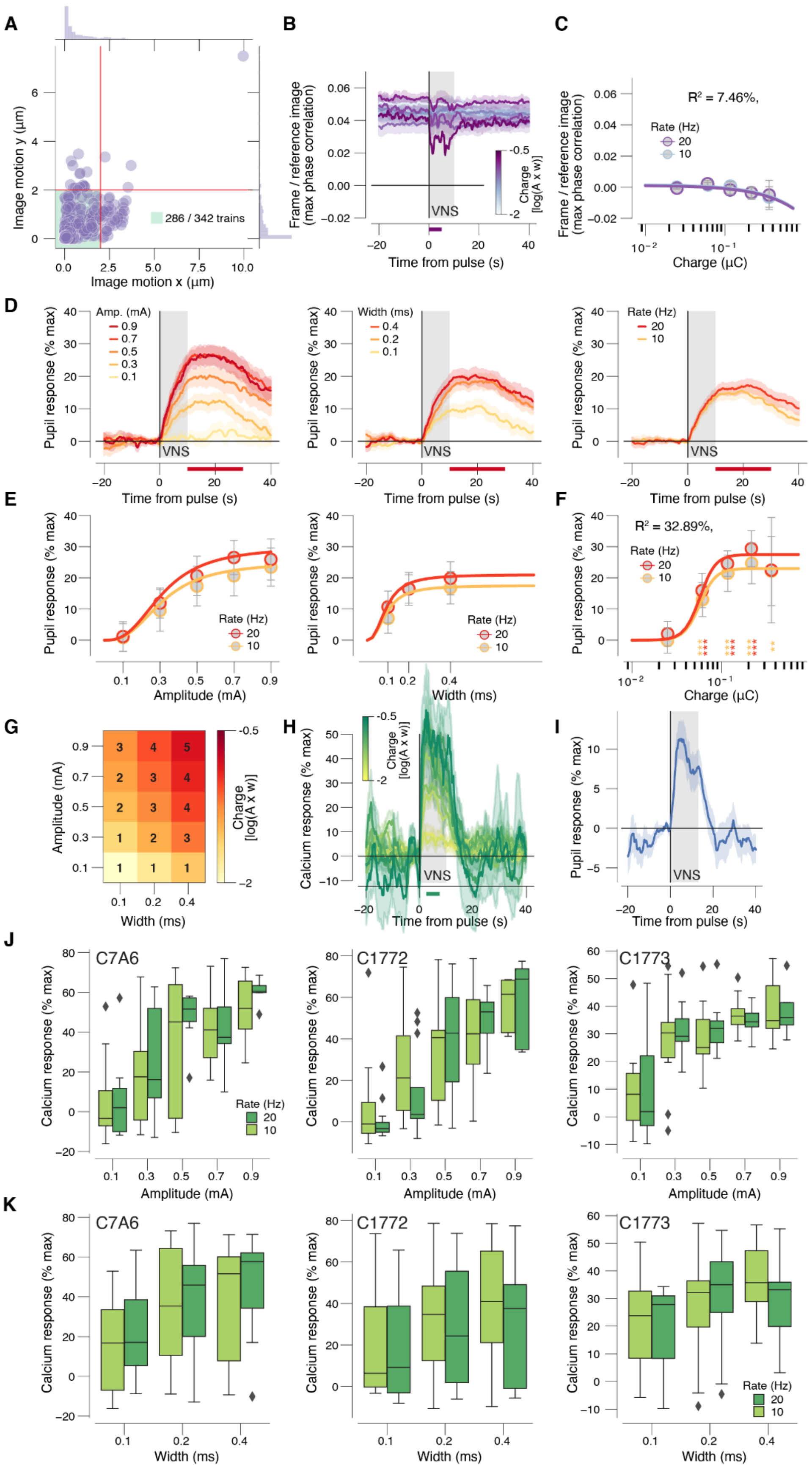
A
B
C
D
E
F
G
H
I
J
K
Image motion y (μm)
Image motion x (μm)
286 / 342 trains
Frame / reference image
(max phase correlation)
Time from pulse (s)
VNS
Charge
[log(A x w)]
R^2 = 7.46%,
Rate (Hz)
20
10
Charge (μC)
Pupil response (% max)
Amp. (mA)
0.9
0.7
0.5
0.3
0.1
Width (ms)
0.4
0.2
0.1
Amplitude (mA)
R^2 = 32.89%,
Calcium response (% max)
C7A6
C1772
C1773

Supplementary Figure 6. (A) Scatter plot of estimated image motion (from Suite2p's rigid registration step; Methods) in x and y. Data points, VNS pulses; green window, analysis inclusion zone (Methods). **(B)** Correspondence between co-registered frame and the reference image time-locked to VNS separately for pulse charge and collapsed across pulse rate. Grey window, VNS train; purple bar, interval for VNS-evoked correspondence measures (Methods); data are presented as mean values ± s.e.m. (across VNS events). **(C)** VNS-evoked correspondence measures separately per pulse charge bin and pulse rate. Colored lines, fitted log logistic function (Methods); data are presented as mean values ± 1.96 × s.e.m. (across VNS events); stats, two-sided one sample t-test (tested against 0; ***$p < 0.001$, **$p < 0.01$; *$p < 0.05$ false discovery rate corrected). **(D)** VNS-evoked pupil responses separately for pulse amplitudes (left), widths (middle) and rates (right) collapsed across two other stimulation parameters. Grey window, VNS pulse (10 s); red bars, interval for VNS-evoked pupil response measures (Methods); data are presented as mean values ± s.e.m. (across VNS events). **(E)** VNS-evoked pupil response measures separately for pulse amplitudes and rates (collaped across widths, left) and separately for pulse widths and rates (collapsed across amplitudes, right). Colored lines, fitted log logistic function (Methods); data are presented as mean values ± 1.96 × s.e.m. (across VNS events). **(F)** As C, but for VNS-evoked pupil responses as a function of charge bin. **(G)** Grid indicating the five equal size charge bins used to reduce the parameter space in imaging experiments. **(H)** VNS-evoked calcium time-courses. Each trace is a charge/pulse bin and train rate. Grey window, VNS train; green bar, interval for VNS-evoked calcium response scalar measures (see Methods); data are presented as mean values ± s.e.m. (across VNS events). **(I)** VNS-evoked pupil responses in pupil luminance control experiment (Methods). **(J)** VNS-evoked calcium response scalars plotted separately per animal, and separately for pulse amplitudes and rates (collapsed across widths). Box plots indicate the median (center line), first quartiles (box edges), minimum/maximum values (whiskers), and outliers (diamonds; based on interquartile range). **(K)** As J, but for for pulse widths and rates (collapsed across amplitudes). All panels except G,I: N=12 repetitions for each unique parameter combination (before artefact rejection; Methods). Panel I: N=120 repetitions of the same parameter combination (before artefact rejection; Methods). Source data are provided as a Source Data file.

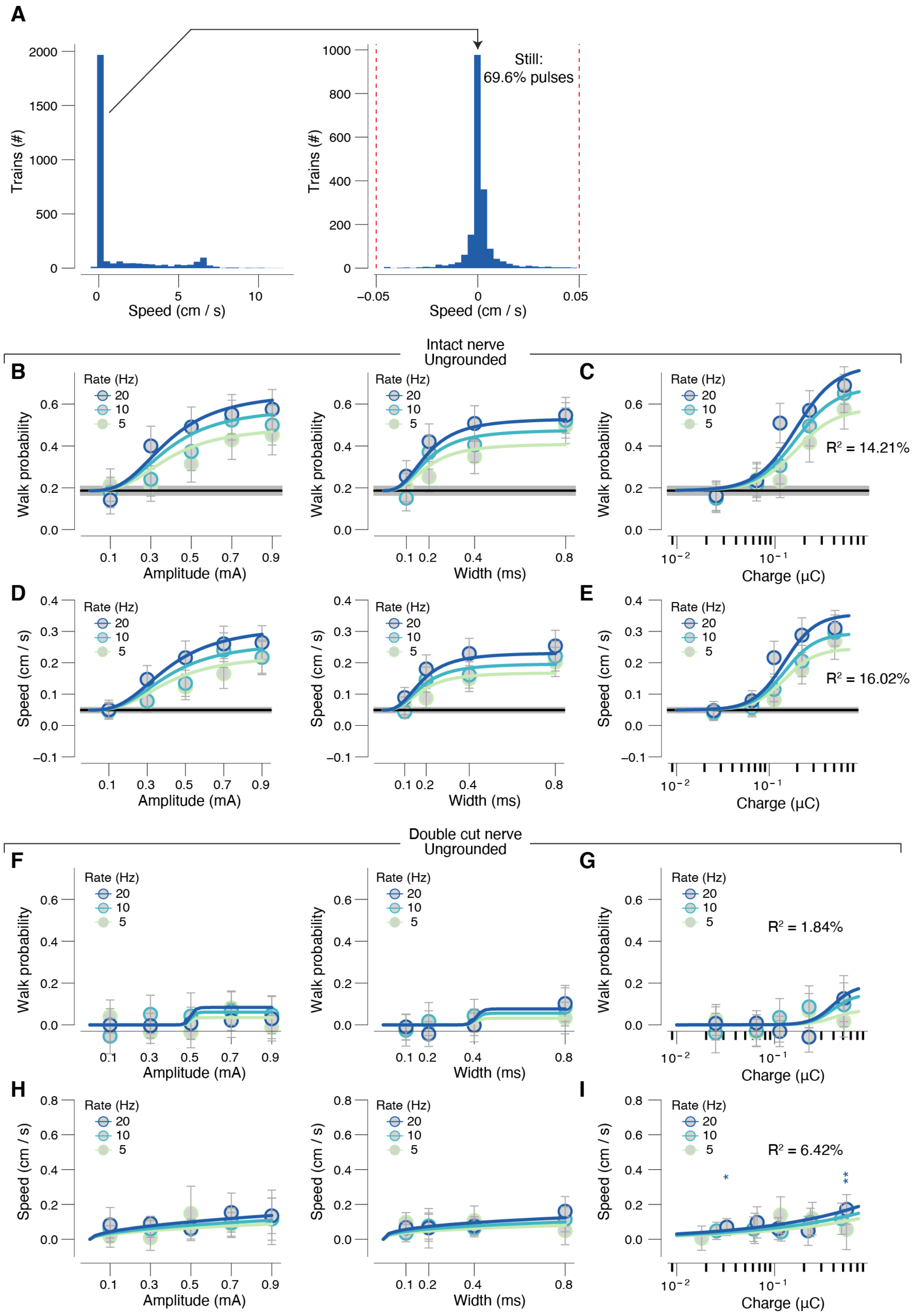
A
Still:
69.6% pulses
Trains (#)
Speed (cm / s)
Intact nerve
Ungrounded
B
C
Rate (Hz)
20
10
5
Walk probability
Amplitude (mA)
Width (ms)
Charge (μC)
R² = 14.21%
D
E
Speed (cm / s)
R² = 16.02%
Double cut nerve
Ungrounded
F
G
R² = 1.84%
H
I
R² = 6.42%

Supplementary Figure 7. (A) Left: histogram of VNS-evoked walking speed in 'parameter exploration experiment' (intact nerve, ungrounded). Right: as left, but zoomed in on the large peak around zero walking speed. Dashed red line, cutoff for defining walking. N=10 (total of 45 sessions). **(B)** VNS-evoked walk probability (without correction for reversion to the mean; see Methods) separately for pulse amplitudes and rates (collapsed across widths, left) and separately for pulse widths and rates (collapsed across amplitudes, right). Black line, spontaneous walk probability in pre-stimulation baseline interval; colored lines, fitted log logistic function (Methods); data are presented as mean values ± 1.96 × s.e.m. (across VNS events). **(C)** As B, but for charge bins. **(D)** VNS-evoked walking speed (on all trials; without correction for reversion to the mean; see Methods) separately for pulse amplitudes and rates (collapsed across widths, left) and separately for pulse widths and rates (collapsed across amplitudes, right). Black line, spontaneous walk probability in pre-stimulation baseline interval; colored lines, fitted log logistic function (Methods); data are presented as mean values ± 1.96 × s.e.m. (across VNS events). **(E)** As D, but for charge bins. **(F)** VNS-evoked walk probability in grounded animals (see Methods) separately for pulse amplitudes and rates (left) and separately for pulse widths and rates (right). Colored lines, fitted log logistic function (Methods); data are presented as mean values ± 1.96 × s.e.m. (across VNS events). **(G)** As A, but for charge bins. Stats, two-sided one sample t-test (tested against 0; ***$p < 0.001$, **$p < 0.01$; *$p < 0.05$ false discovery rate corrected). **(H,I)** As F,G, but for walking speed on walk trials only. Panels A-D: N=27 repetitions for each unique parameter combination (before artefact rejection; Methods). Panels F-I: N=24 repetitions for each unique parameter combination (before artefact rejection; Methods). Source data are provided as a Source Data file.

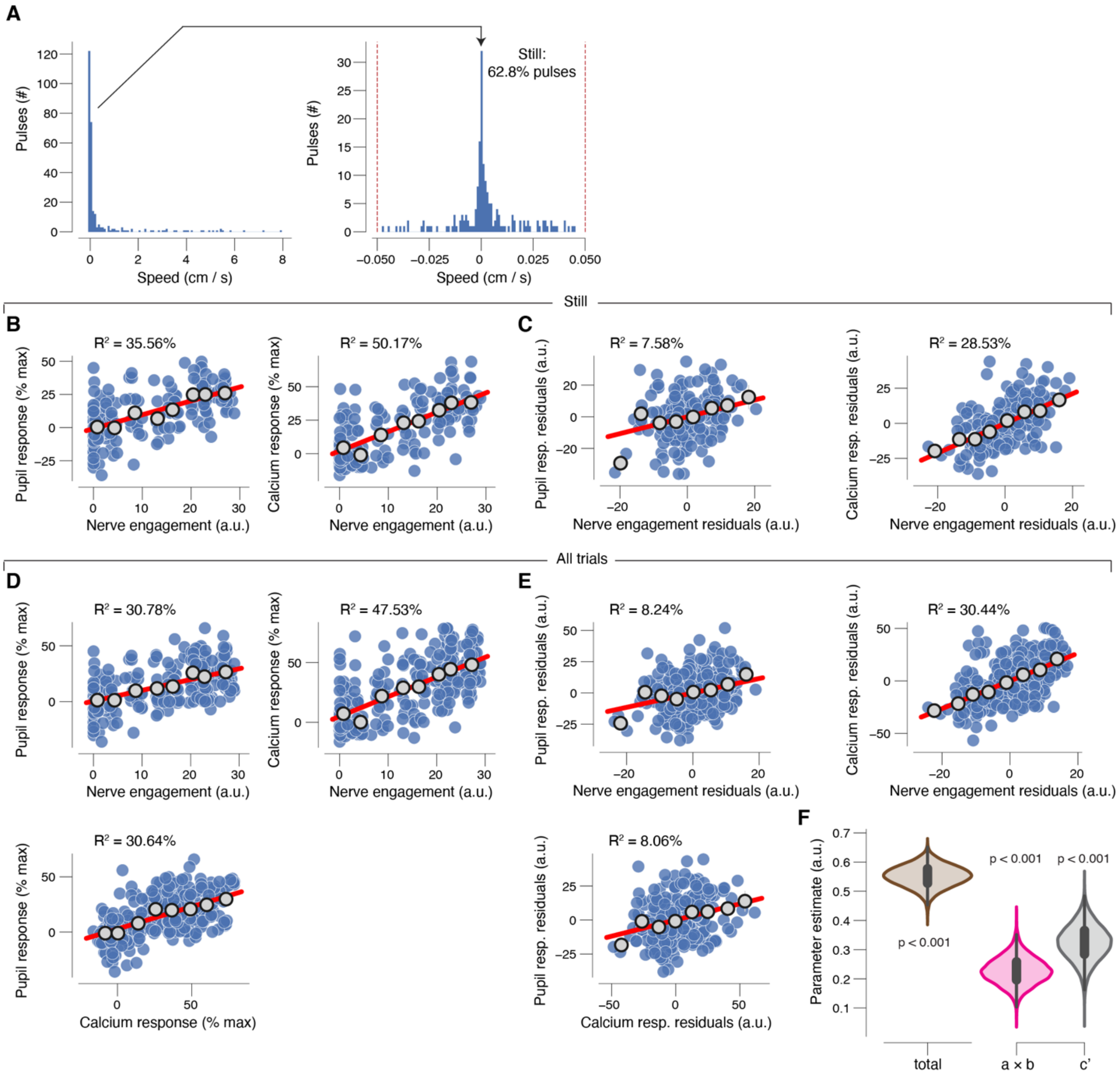

Supplementary Figure 8. (A) Left: histogram of VNS-evoked walking speed in the 'axon imaging experiment'. Right: as left, but zoomed in on the large peak. Dashed red line, cutoff for defining walking. **(B)** Scatterplot of the relationship between VNS-evoked pupil responses (left) or calcium responses (right) and VNS in absence of walking. Blue data points are individual VNS trains; grey data points are nerve engagement defined bins (equal size); red line, linear fit, Pearson correlation, $p < 0.001$. **(C)** As B, but after removing effects of VNS-evoked calcium responses (left) or pupil responses (right) (partial correlation). Pearson correlation, $p < 0.001$. **(D,E)** As B,C, but for all trials, irrespective of walking. **(F)** For all trials, irrespective of walking: fitted regression coefficients (kernel density estimate of 5K bootstrapped replicates) of the total effect (brown), indirect path (mediation; pink) and the direct path (grey). Stats, fraction of bootstrapped coefficients smaller than 0. Panels B-E: sequential polynomial regression (Methods) indicated that in all cases a 1st-order (linear) fit was superior to a constant fit, and that a 2nd-order (quadratic) fit was not superior to the first-order fit. All panels: N=12 repetitions for each unique parameter combination (before artefact rejection; Methods). Source data are provided as a Source Data file.
