# Supplementary material for: Graded recruitment of pupil-linked neuromodulation by parametric stimulation of the vagus nerve
Source: Nat Commun. 2021 Mar 9;12:1539. doi: 10.1038/s41467-021-21730-2 (PMC7943774; doi:10.1038/s41467-021-21730-2)
Supplement: Supplementary file 3 — Reporting Summary [file 41467_2021_21730_MOESM3_ESM.pdf]

nature research

Corresponding author(s): Matthew J. McGinley

Last updated by author(s): Nov 10, 2020

Reporting Summary

Nature Research wishes to improve the reproducibility of the work that we publish. This form provides structure for consistency and transparency in reporting. For further information on Nature Research policies, see our Editorial Policies and the Editorial Policy Checklist.

Statistics

For all statistical analyses, confirm that the following items are present in the figure legend, table legend, main text, or Methods section.

n/a	Confirmed	
☐	☒	The exact sample size (n) for each experimental group/condition, given as a discrete number and unit of measurement
☐	☒	A statement on whether measurements were taken from distinct samples or whether the same sample was measured repeatedly
☐	☒	The statistical test(s) used AND whether they are one- or two-sided<br>*Only common tests should be described solely by name; describe more complex techniques in the Methods section.*
☒	☐	A description of all covariates tested
☐	☒	A description of any assumptions or corrections, such as tests of normality and adjustment for multiple comparisons
☐	☒	A full description of the statistical parameters including central tendency (e.g. means) or other basic estimates (e.g. regression coefficient) AND variation (e.g. standard deviation) or associated estimates of uncertainty (e.g. confidence intervals)
☐	☒	For null hypothesis testing, the test statistic (e.g. F, t, r) with confidence intervals, effect sizes, degrees of freedom and P value noted<br>*Give P values as exact values whenever suitable.*
☒	☐	For Bayesian analysis, information on the choice of priors and Markov chain Monte Carlo settings
☒	☐	For hierarchical and complex designs, identification of the appropriate level for tests and full reporting of outcomes
☐	☒	Estimates of effect sizes (e.g. Cohen's d, Pearson's r), indicating how they were calculated

Our web collection on statistics for biologists contains articles on many of the points above.

Software and code

Policy information about availability of computer code

Data collection	LabVIEW 2016, ScanImage 2016, MATLAB 2016
Data analysis	Python 3.7; DeepLabCut 2.1.8.2, Suite2p 0.7.1; MNE 0.19.2; R 3.6.0; lavaan 0.6-7. Custom Python code is available on https://doi.org/10.5281/zenodo.4243062.

For manuscripts utilizing custom algorithms or software that are central to the research but not yet described in published literature, software must be made available to editors and reviewers. We strongly encourage code deposition in a community repository (e.g. GitHub). See the Nature Research guidelines for submitting code & software for further information.

Data

Policy information about availability of data

All manuscripts must include a data availability statement. This statement should provide the following information, where applicable:

- Accession codes, unique identifiers, or web links for publicly available datasets
- A list of figures that have associated raw data
- A description of any restrictions on data availability

The data and the pre-trained DeepLabCut network are publicly available on https://doi.org/10.6084/m9.figshare.12899375.

Field-specific reporting

Life sciences study design

All studies must disclose on these points even when the disclosure is negative.

Sample size	No sample-size calculation was performed. Rather, we based our sample size on previous work of VNS stimulation in rodents (Hulsey et al., Exp. Neurology, 2017), and simultaneous cholinergic axon imaging and pupillometry in mice (Reimer & McGinley et al., Nat Comm, 2016).
Data exclusions	In the 'parameter exploration' experiment, we excluded VNS events from the analyses for which we could not reliably record pupil size (due to blinking or other reasons; 1.51% of events).<br><br>In the 'baseline pupil dependence experiment, we excluded 8 (out of 23) sessions in which the mean VNS-evoked pupil response was smaller than 1% signal change. Of the remaining sessions, we excluded VNS events from the analyses for which we could not reliably record pupil size (due to blinking or other reasons; 1.92% of events).<br><br>In the 'axon imaging experiment', we performed a total of 31 imaging sessions in three unique mice. Of these, 12 imaging sessions were successful (three, four and five sessions per mouse, respectively). We excluded the remaining sessions from the analyses based on three criteria: (i) no observable axons after preprocessing (4 sessions), (ii) more than 2 μm image motion on more than 10 trials (see also Analysis of calcium imaging data, below) (one session), and (iii) overall VNS-evoked activity in less than 750 pixels (see also Analysis of calcium imaging data, below) (19 sessions). Of the remaining sessions, we excluded VNS events from the analyses for which we could not reliably record pupil size and/or observed more than 2 μm motion in either x or y direction (16.67% of events).
Replication	All experiments were repeated in multiple animals, verifying that results were reproducible. Ten ungrounded animals with intact nerve were part of the 'parameter exploration experiment', which resulted in a total of 45 independent replications of each unique VNS parameter combination. Three ungrounded animals with intact nerve were part of the 'axon imaging experiment', which resulted in a total of 12 independent replications of each unique VNS parameter.
Randomization	There were no different experimental groups.
Blinding	Blinding was not relevant in this study, as there were no different experimental groups. Rather, each animal was exposed to all unique combinations of VNS parameters.

Reporting for specific materials, systems and methods

We require information from authors about some types of materials, experimental systems and methods used in many studies. Here, indicate whether each material, system or method listed is relevant to your study. If you are not sure if a list item applies to your research, read the appropriate section before selecting a response.

Materials & experimental systems

n/a	Involved in the study
☒	☐ Antibodies
☒	☐ Eukaryotic cell lines
☒	☐ Palaeontology and archaeology
☐	☒ Animals and other organisms
☒	☐ Human research participants
☒	☐ Clinical data
☒	☐ Dual use research of concern

Methods

n/a	Involved in the study
☒	☐ ChIP-seq
☒	☐ Flow cytometry
☒	☐ MRI-based neuroimaging

Animals and other organisms

Policy information about studies involving animals; ARRIVE guidelines recommended for reporting animal research

Laboratory animals	We used a cross of two transgenic lines for the imaging experiments, and wild-type mice in all other experiments. The wild-type mice (N=31; all male) were of C57BL/6 strain (Jackson Labs). The mice for cholinergic axon imaging (N=3; 2 female; age, 8–18weeks) were heterozygous for the ChAT-cre (Jackson labs strain B6; 129S6-Chattm1(cre)Lowl/J) and Ai162 GCaMP6 reporter lines (Allen Institute). Mice received ad libitum food and water and were individually housed after VNS cuff implantation. Mice were housed under standard conditions (temperatures of 65-75°F with 40-60% humidity) in ventilated racks on a regular light-dark cycle, and all experimental manipulations were done during the light phase.
Wild animals	No wild animals were used in the study.
Field-collected samples	No field collected samples were used in the study.
Ethics oversight	Institutional Animal Care and Use Committee (IACUC) of Baylor College of Medicine.

Note that full information on the approval of the study protocol must also be provided in the manuscript.
